# miRNA Involvement in Cerebral Ischemia-Reperfusion Injury

**DOI:** 10.3389/fnins.2022.901360

**Published:** 2022-06-10

**Authors:** Maria-Adriana Neag, Andrei-Otto Mitre, Codrin-Constantin Burlacu, Andreea-Ioana Inceu, Carina Mihu, Carmen-Stanca Melincovici, Marius Bichescu, Anca-Dana Buzoianu

**Affiliations:** ^1^Department of Pharmacology, Toxicology and Clinical Pharmacology, Iuliu Haţieganu University of Medicine and Pharmacy, Cluj-Napoca, Romania; ^2^Faculty of Medicine, Iuliu Haţieganu University of Medicine and Pharmacy, Cluj-Napoca, Romania; ^3^Department of Morphological Sciences, Iuliu Haţieganu University of Medicine and Pharmacy, Cluj-Napoca, Romania

**Keywords:** miRNAs, ischemia reperfusion, cell death, inflammation, oxidative stress

## Abstract

Cerebral ischemia reperfusion injury is a debilitating medical condition, currently with only a limited amount of therapies aimed at protecting the cerebral parenchyma. Micro RNAs (miRNAs) are small, non-coding RNA molecules that *via* the RNA-induced silencing complex either degrade or prevent target messenger RNAs from being translated and thus, can modulate the synthesis of target proteins. In the neurological field, miRNAs have been evaluated as potential regulators in brain development processes and pathological events. Following ischemic hypoxic stress, the cellular and molecular events initiated dysregulate different miRNAs, responsible for long-terming progression and extension of neuronal damage. Because of their ability to regulate the synthesis of target proteins, miRNAs emerge as a possible therapeutic strategy in limiting the neuronal damage following a cerebral ischemic event. This review aims to summarize the recent literature evidence of the miRNAs involved in signaling and modulating cerebral ischemia-reperfusion injuries, thus pointing their potential in limiting neuronal damage and repair mechanisms. An in-depth overview of the molecular pathways involved in ischemia reperfusion injury and the involvement of specific miRNAs, could provide future perspectives in the development of neuroprotective agents targeting these specific miRNAs.

## Introduction

Stroke represents the third leading cause of death and a major debilitating medical condition. It is responsible for permanent disabilities in approximately 80% of post-stroke patients (Moskowitz et al., 2010; Lallukka et al., 2018). Metabolic disruption of neurons activates immune responses, resulting in a complex chain of molecular events, which further promote progressive cellular damage and irretrievable neuronal death (Moskowitz et al., 2010; Khoshnam et al., 2017).

The ischemic/reperfusion (I/R) injury is caused by a sudden restriction of blood supply and oxygen, followed by subsequent restoration of blood flow and reoxygenation, contributing supplementary to the global oxidative stress (Eltzschig and Eckle, 2011). The I/R injury is the main actor in the neuroinflammatory repertoire, triggering different cell death provoking events, which include apoptosis, blood-brain barrier (BBB) disruption and mitochondrial dysfunction (Eltzschig and Eckle, 2011; Khoshnam et al., 2017).

The neuroprotective agents under current research address either the ischemic core, or the viable penumbra region, with the aim of reestablishing the collateral blow flow and ameliorating the microenvironment damaged tissue (Eltzschig and Eckle, 2011; He et al., 2021). The standard therapeutic strategy for ischemic stroke remains thrombolytic reperfusion therapy provided by intravenous tissue plasminogen activator that is, however, limited by a short therapeutic window of 3-4,5 hours (Del Zoppo et al., 2009; IST-3 collaborative group et al., 2012; Fonarow et al., 2014).

Preclinical translation of neuroprotective drugs into clinical settings is failing. Even with advancing experimental studies on animal models, with excellent human reproducibility provided by thromboembolic stroke models, i.e., reproducible infarct size, and penumbra zone, there are still many promising neuroprotective agents in preclinical studies that fail to show a significant effect on patients (Dirnagl, 2006; Canazza et al., 2014; Luo et al., 2019). Dirnag et al. attributed this limited clinical potential of experimental drugs to statistical errors, lack of blinding and randomization of the animals, and negative publication bias (Dirnagl, 2006). Unexplored impediments steam from the limited ability of drugs to penetrate the BBB and target the ischemic neuronal tissue, resulting in decreased efficient concentration of the neuroprotective agents (Saugstad, 2010; Ponnusamy and Yip, 2019). In this context, selective drug delivery systems such as stroke tissue-related homing peptides and nanoparticles-mediated agents are emerging (Hong et al., 2008; He et al., 2021).

Micro RNAs (miRNAs) are small, non-coding RNA molecules, containing around 18–25 nucleotides, which pose a post-transcriptional regulatory role by down-regulating messenger RNAs (mRNAs) (Jonas and Izaurralde, 2015). Binding to the target mRNAs by base pairing, miRNAs negatively regulate gene expression of mRNAs *via* cleavage of mRNA, translation repression or destabilization of mRNA structure (Bartel, 2009; MacFarlane and Murphy, 2010).

The first pathological condition described, related to miRNAs was chronic lymphocytic leukemia (Calin et al., 2004). Since then, multiple studies outline the potential of miRNAs to mediate several pathological mechanisms of human diseases—i.e., cancer, neurological disorders, immune system disorders, acting as signaling molecules and mediators of cell-cell communication in different cellular processes such as proliferation, differentiation, and apoptosis (Smirnova et al., 2005; Garofalo et al., 2010; Tüfekci et al., 2014). MicroRNAs are key master regulators of gene expression in the brain, in processes related to brain development and its normal functioning, i.e., synaptogenesis, myelination, cerebral vasculogenesis and angiogenesis, but also in different brain disorders: ischemic stroke, neurodegenerative disease, traumatic brain injury, spinal cord injury, hypoxic-ischemic encephalopathy (Saugstad, 2010; Ponnusamy and Yip, 2019).

MicroRNAs also play a pivotal role in I/R injury, the main contributor to reactive oxygen species (ROS) production, cellular metabolic disfunctions associated with/underlying ischemic stroke (Ouyang et al., 2015; Cao et al., 2021). Recent studies have shown that I/R-related miRNAs alter the mitochondrial response and mediate multiple pathways that further promote neuronal survival and apoptosis (Jeyaseelan et al., 2008; Di et al., 2014; Hu et al., 2015; Ouyang et al., 2015). Min et al. highlighted the altered expression profile of miRNAs in brain I/R injury, which consisted of 15 miRNAs upregulated and 44 miRNAs downregulated (Min et al., 2015). MiRNAs modulate critical signaling pathways in I/R injury, associated with fibrosis, neoangiogenesis, necrosis, apoptosis and inflammation (Ghafouri-Fard et al., 2020).

However, miRNAs have also been reported in promoting the pathogenesis of ischemic stroke—i.e., atherosclerosis, hypertension, hyperlipidemia, plaque rupture, bidirectionally influencing the pathological chain of ischemic events, both pathogenesis and pathways (Rink and Khanna, 2011). In this direction, advancing the knowledge in gene functions using agomirs or antagomirs—double stranded miRNA agents, chemically modified at antisense strand that act as miRNA mimickers or inhibitors—could provide potential neuroprotective effects in modulating pathological processes in ischemic injuries (Kadir et al., 2020).

Neuroscience confronts limited therapeutic strategies aimed at protecting ischemic tissue, for which there is a critical and urgent need for advancing our knowledge. A depth overview of the molecular pathways involved in ischemic stroke, which are targeted by specific miRNAs, could provide future perspectives in the development of neuroprotective miRNA agents. This review aims to summarize the recent literature evidence of the miRNAs involved in signaling and modulating cerebral ischemia-reperfusion injuries, thus pointing their potential in limiting neuronal damage and repair mechanisms.

## miRNAs in Neurological Diseases

Development of the adult brain and its functions are a highly studied subject in today’s literature. Normal brain development proceeds *via* complex multistep processes, which involves early embryonic stage- neurogenesis, consisting in proliferation and differentiation of precursor neuronal cells, continuing to myelination and synaptogenesis in the childhood and adulthood period, which contributes to synaptic plasticity and memory (Semple et al., 2013). MiRNAs play essential roles in controlling neurodevelopment processes and normal brain functions, and dysregulation of miRNA expression profiling has been related to perinatal brain injury (Cho et al., 2019). Ponnusamy and Yip (2019) deciphered the role of miRNA involved in normal brain development’ processes under normoxic and hypoxic conditions, consisting in myelination, axonal outgrowth, dendric outgrowth, synaptogenesis, neuronal differentiation, neuronal migration, angiogenesis.

Neurodegenerative diseases, which are mainly characterized by intracellular or extracellular protein aggregate formation, resulting to neuron dysfunction in certain brain areas, includes Alzheimer’s disease (AD), Parkinson’s disease (PD), Huntington’s disease and multiple sclerosis (MS) (Quinlan et al., 2017).

Mounting evidence suggested the role of miRNAs-based therapeutics in modulating the prognosis of neurodegenerative diseases, emerging new miRNAs biomarkers for a better disease control (Quinlan et al., 2017). Thus, Juźwik et al. (2019) in a systematic review of 12 neurodegenerative disease identified 10 miRNAs frequently dysregulated, including miR-9-5p, miR-21-5p, miR-29a-3p, miR-29b-3p, miR-29c-3p, miR-124-3p, miR-132-3p, miR146a-5p, miR-155-5p, and miR-223-3p. Notably, a different expression level of miRNAs, miR-9-5p, miR-21-5p, the miR-29, miR-124-3p, and miR-132-3p have been revealed, suggesting the mixed expression levels of miRNAs.

PD is characterized by dopaminergic neuron loss from the substantia nigra, with dysregulated level of miRNAs expression in the striatal brain areas and dopaminergic neurons (Nies et al., 2021). Prefrontal cortex of post-mortem PD patients exhibited 125 dysregulated miRNAs, of which miR-10b-5p levels being associated with clinical onset in both PD and Huntington’s Disease (Hoss et al., 2016). The pathogenesis of PD related to miRNAs have been explained by modulation of PD-associated genes and protein expression related to α-synuclein-induced neuroinflammation, and degeneration of dopaminergic neurons (Nies et al., 2021). Down regulation of miR-425 in MPTP injected mouse PD model contributes to necroptosis and apoptosis activation, disintegration of mitochondrial membrane, ultimately leading to neuron loss and dopamine depletion. Moreover, miR-103a-3p, miR-30b-5p, and miR-29a-3p exhibited high levels of expression after Levodopa treatment, suggesting the role of miRNAs as disease modifier agents in PD (Serafin et al., 2015). Recent studies have shown that suppressing miR-34a can improve neuronal loss related to PD (Chua and Tang, 2019).

Sun et al. (2021) using bioinformatic analysis, reviewed the dysregulated miRNAs expression profiling in tissues of AD patients’ brain, blood and CSF, correlated with pathological processes. Therefore, 27 dysregulated miRNAs identified have been related to neuroinflammation, amyloidogenesis, tau phosphorylation, synaptogenesis, apoptosis, and neuron degradation (Sun et al., 2021).

Multiple *in vivo* and *in vitro* animal models revealed the potential of miRNAs to counteracting beta-amyloid or tau reduction, inhibiting of apoptosis, and synaptic protection. In APP/PS1 transgenic mice, miR-137 exhibited reduced levels in the cerebral cortex, hippocampus, and serum, suggesting the neuroprotective potential of miR-137 to suppress p-tau overexpression (Jiang et al., 2018b). Moreover, inhibition of miR-98 in N2a/APP cells suppressed Aβ production by upregulating insulin-like growth factor 1 pathway (Hu et al., 2013, 1).

Neuroinflammation plays critical roles in MS pathogenesis consisting in dysregulation of inflammatory cell events in the brain, resulting in BBB disruption, damage of myelin and oligodendrocytes, neuro-axonal damage and inflammation (Haase and Linker, 2021).

MiR-155 which exhibited upregulated levels in MS, poses important role in BBB disruption under inflammatory conditions, which drives to demyelination processes, i.e., microglial activation, polarization of astrocyte. In 58 MS patients with adult onset, miR-320a, miR-125a-5p, miR-652-3p, miR-185-5p, miR-942-5p, miR-25-3p were significantly upregulated in peripheral blood samples, controlling transcription factors of SP1, NF-κB, TP53, HDAC1, and STAT3 (Nuzziello et al., 2018).

Unbalance of inflammatory reactions including dysfunction of memory T-cells and Treg cells contributed to continuous and progression inflammatory demyelinating of CNS. For instance, in MS patients, miR-19a, miR-19b, miR-25, and miR-106 elicited significantly upregulated levels in Treg cells compared with healthy controls (Gao et al., 2021). Targeting dysregulated miRNAs represents a therapeutic strategy. Thus, inhibiting let-7e decrease the differentiation of Th1 and Th17 cells, reducing the severity of MS in experimental autoimmune encephalomyelitis (Angelou et al., 2019). Increasing evidence ascertained the involvement of miRNAs in the initiation and progression of multifold types of cancer. Petrescu et al. (2019) reviewed the main dysregulated miRNAs related to brain tumors pathogenesis in glioma, meningioma, pituitary adenoma, and astrocytoma.

Multiple pathological processes associated with gliomagenesis were controlled by miRNAs. From disrupting BBB by targeting junctional proteins, zonula occludens-1 (ZO-1), occludin and β-catenin, to angiogenic, infiltration and migration of glioma cells by downregulating MMP2, MMP9, VEGF, all these tumor promoting processes are modulated by several miRNAs (Petrescu et al., 2019).

MiRNAs could be also used as clinical prognosis biomarkers. In 90 serum astrocytoma patients, miR-15b-5p, -16-5p, -19a-3p, -19b-3, 20a-5p, 106a-5p, 130a-3p, 181b-5p and 208a-3p exhibited upregulation levels, with miR-19a-3p, -106a-5p, and -181b-5p being associated with lower survival rate (Zhi et al., 2015).

## Cerebral Ischemia/Reperfusion Injuries

### Histopathological Findings in Hypoxic/Ischemic Brain Injury

Hypoxic or ischemic brain injury give rise to a heterogeneity of histological findings, in which the neurons, the glial cells, the neuropile and the brain microvasculature are affected. These alterations in brain histological structures occur in chronological order and depends on the magnitude and duration of ischemia, and the extension of tissue damage. Two areas are examined: the ‘’ischemic core” or the irreversibly damaged area, and the ‘’ischemic penumbra,” the hypoperfused area, which still contains viable cells.

#### Neurons and Glial Cells Modifications

The earliest change which occurs in the ischemic core is represented by neuronal swelling, because of the cytotoxic edema caused by ion alteration. The damaged neurons are large, with pale staining cytoplasm and pyknotic nucleus in hematoxylin and eosin (H&E) staining. After hours, in the ischemic core appear the red, eosinophilic, or ischemic neurons, characterized on routine histological sections by cell shrinkage, a pyknotic nucleus without nucleolus, and a highly eosinophilic cytoplasm, devoided of Nissle bodies. These neurons may be found also in the penumbra area for 1 or 2 days. Another aspect of advanced neuronal degeneration is represented by ‘ghost neurons’, found in the ischemic core and in the ischemic penumbra zone, which exhibits an irregular and very ill-defined cell border, pale staining cytoplasm in H&E staining and pyknotic, dark nucleus. The disintegration of dead neurons leads to parenchymal necrosis and release of cellular debris, which later will be engulfed by macrophages (Mărgăritescu et al., 2009; Rahaman and Del Bigio, 2018).

Activation and proliferation of microglia, the resident macrophages in the central nervous system, occurs in the ischemic core in the first hours after ischemic injury, being involved in removing the necrotic tissue. During activation, microglia undergo morphological changes, with increase in cell body size and retraction of cytoplasmatic processes, acquiring an amoeboid phenotype in the ischemic core. In the ischemic penumbra and in the marginal zone we can find numerous highly ramified microglia (reactive microglia), which can migrate to the ischemic core, suggesting the fact that microglia may exhibit different morphological patterns, according to degree of ischemia and the time interval after ischemia (Zhang, 2019). After about 3 days, a lot of bone marrow-derived macrophages infiltrated the ischemic core and the ischemic penumbra (mostly), where they phagocytose the cellular and myelin debris, having a foamy appearance on histological sections. Activated microglia express high levels of immunomarker Iba1 +, while bone marrow-derived macrophages are highly positive for CD45 (Mărgăritescu et al., 2009; Li et al., 2014b; Magaki et al., 2018; Washida et al., 2019; Zhang, 2019).

In the ischemic core, swelling or edematous astrocytes may be found in the early phase, with a pale staining cytoplasm and disrupted cytoplasmatic processes; eventually, these cells will die. In the ischemic penumbra, the surviving astrocyte proliferate and undergo hypertrophy (reactive astrogliosis), expressing high amounts of glial fibrillary acidic protein. In routine histological sections, reactive astrocytes are large, star-shaped cells, having a coarse nuclear chromatin, glassy eosinophilic cytoplasm and long, branching cytoplasmatic processes; they are also called gemistocytic astrocytes. Astrogliosis represents a hallmark of nervous tissue injury after ischemia, and always follows the microglial activation and blood-derived macrophages invasion. After several days, the astrocytes and microglial cells from the ischemic penumbra surround the ischemic core and the cells will fill the necrotic areas, forming the glial scar tissue, an eosinophilic zone in H&E staining, with neuron loss and numerous glial cells, mainly reactive astrocytes (Mărgăritescu et al., 2009; Li et al., 2014b; Magaki et al., 2018).

In the first hours after ischemic injury, oligodendrocytes damage may cause axonal degeneration and demyelination, leading to rarefaction of the white matter (Mărgăritescu et al., 2009; Washida et al., 2019).

#### Microvascular Changes

In the ischemic core, structural changes of the small blood vessels are observed, such as: endothelial cell (ECs) swelling, pericyte and ECs detachment from the basement membrane, narrowing of the lumen, hyalinization and vascular wall thickening and sclerosis, with increase amount of collagen fibers and disintegration of vascular smooth muscle cells. These vascular modifications, in addition to morphological changes of astrocyte foot processes, lead to alteration of the BBB, which cause the vasogenic edema in the neuropil. Disruption of BBB or disintegration of capillaries in the necrotic areas, induce the appearance of microhemorrhages, extravasated and lysed erythrocytes releasing hemosiderin pigment, which is phagocytized by macrophages (siderophages) (Mărgăritescu et al., 2009; Rahaman and Del Bigio, 2018; Liu et al., 2019a).

The ischemic penumbra contains congested blood vessels, surrounded by perivascular edema. After 3 days, neovascularization occurs within the ischemic penumbra, but the newly formed blood vessels are abnormal, thin, highly permeable, thus increasing the pre-existing brain edema (Rahaman and Del Bigio, 2018; Liu et al., 2019a).

#### Inflammatory Reaction

Polymorphonuclear leukocytes (PMNs) and macrophages play a key role in early inflammatory reaction after brain ischemia, while lymphocytes (mostly T lymphocytes), are involved in the delayed phases of ischemia. An acute inflammatory reaction appears within the first 4-6 hours after ischemic injury, with PMNs infiltration in the necrotic tissue. Within the first 3 days, activated microglia and blood-derived macrophages invade the necrotic area, engulfing the cellular and myelin debris (lipid-laden macrophages) (Kawabori and Yenari, 2015; Anrather and Iadecola, 2016).

### General Mechanisms of Cerebral Ischemia/reperfusion Injury

Neuronal damage after recanalization has long been known to occur following ischemic stroke through a unique type of injury that is not expressed during the hypoxic period (S.M. Humphrey et al., 1973; Baird et al., 1994). As ischemic events are responsible for stroke in almost 80% of cases, even with the achievement of reperfusion *via* thrombolysis, stent retrievers or spontaneous reperfusion, I/R injuries have been shown to have deleterious and noteworthy effects of brain function and ischemic area after artery occlusion (Zhang et al., 1994). Animal studies have shown that the area damaged by the initial ischemic event can increase in size after repermeabilization of the affected artery, compared to continuous occlusion (Zhang et al., 1994). As pathophysiological mechanism may be possible targets for therapy and prevention of reperfusion injury, altering the BBB has been thought as the main mechanism involved. New evidence suggests multiple damage mechanism that can alter neuronal function in I/R injury such as the activation of the complement system (inhibition of which may yield less ischemia-reperfusion cardiac injury), the increase in leukocyte taxis to the affected area (the depletion of which can be a target in limiting reperfusion damage), cellular component damage, the stress caused by ROS and the activation of platelets can cause reperfusion damage and cerebral edema (Lin et al., 2016; Wu et al., 2018). Another molecular mechanism for brain damage after I/R concerns matrix metalloproteinases (MMPs) and their ability to interrupt endothelial junctions after restoration of blood flow (Candelario-Jalil et al., 2009). The vasogenic edema is caused by a biphasic “opening” of the BBB, with the early phase occurring several hours after reperfusion and being related to the activation of gelatinase A (MMP-2) and the second, 1 to 2 days after restoration of blood flow, associated with the expression and activation of gelatinase B (MMP-9) and stromelysin-1 (MMP3) (Rosenberg and Yang, 2007).

ROS are responsible for the damage to cellular components, such as mitochondria, nucleic acids and proteins (Brieger et al., 2012). Their role in reperfusion injury has long been presumed and recent data confirm that superoxide molecules can be produced after reperfusion following brain ischemia and molecules such as NADPH oxidase (NOX) can be involved in I/R injury in the brain and altering the BBB through their ability to transfer electrons to molecular oxygen (Kim et al., 2017b; Yang, 2019). The latter can be considered a way through which the mechanisms involved in I/R injury link to each other, especially when referring to the first phase of I/R brain injury related to the BBB in case of ischemic brain injury.

An important pathway that can lead to aggravating I/R injury is related to cellular component damage. ROS are causing damage to nucleic acids and macromolecules, as stated above, but also to mitochondria leading to ATP depletion, anaerobic metabolism and malfunctioning of ion pumps (Sanderson et al., 2013). The ischemia-reperfusion model in mitochondrial injury consists of calcium overload due to the altered function of the endoplasmic reticulum, which can generate ROS that may hyperpolarize the mitochondria membrane and surpass the antioxidants present in the cell (Wu et al., 2018). Excess reactive oxygen may escape from the electron transport chain and activate mechanisms that interfere with apoptosis and necrosis, while mitochondrial disfunction regarding fission and fusion becomes impaired during IR injury (Turrens, 2003; Andreyev et al., 2005). Besides an excess in ROS, reperfusion-induced inflammation also causes the release of cytokines, causing cytokine storm that ultimately injures the surrounding tissue (Eltzschig and Eckle, 2011).

Oxidative stress during I/R injury is thought to be caused by three different systems: xanthine oxidase system, NADPH oxidase (NOX) system and nitric oxide synthase (NOS) system (Cantu-Medellin and Kelley, 2013; Ma et al., 2017b). NOX-derived free oxygen radicals are known to cause the increase in local inflammatory cell presence and may lead to impaired perfusion of multiple organs (Sedeek et al., 2009; Meza et al., 2019). Even though the NOS system has a well-established role in providing nitric oxide as an antioxidant protective agent against I/R injury, it is also known that this type of injury can transform NOS into a superoxide generating system, with a resulting decrease in cellular NO and increase in ROS (Forstermann and Munzel, 2006). The free oxygen radicals can promote inflammation in the affected cells and can lead to cellular death (Lisa and Bernardi, 2006).

Inflammation represents a mechanism that has important implications in determining the amount of damage during reperfusion injury. This mechanism can yield effects through the cytokines, and molecules produced by the endothelium and parenchymal cells during I/R injury, but also by the number of leukocytes attracted to the damaged area. Oxidative stress, as mentioned above, can also be a means of aggravating ROS induced inflammation by increasing the expression of pro-inflammatory factors such as TNF-α and interleukin (IL)-1β (Turovsky et al., 2021). The adhesion of white blood cells to the endothelium, slow-rolling and trans-endothelial migration are augmented by flow restoration after ischemia, together with increased oxygen content. As more free oxygen radicals are produced, and leukocyte activation is ongoing due to danger signals, NADPH oxidase produces more ROS, neutrophils are able to release different cell damaging hydrolytic enzymes and generate hypochlorous acid *via* the activity of myeloperoxidase, pore-forming molecules being produced in the detriment of the vascular and parenchymal cells (Granger et al., 1993; Frangogiannis, 2015). Oxidative stress and NO depletion are also responsible for triggering humoral response to I/R injury as molecules such as TNF-α, IL-1, ANG II, LTB4 and PAF (linking the activation of platelets to neutrophil I/R damage) (García-Culebras et al., 2019). In addition to inflammation, complement system activation (C’) has been associated to I/R injury, both by increasing chemotaxis and activation in damage area leukocytes and activating the membrane attack complex to induce cellular damage (Gorsuch et al., 2012). Inhibiting the C5a fragment has also been shown to decrease neutrophile tissue infiltration (Wood et al., 2020). As inflammation is strongly linked to multiple types of cell death, nuclear factors that stimulate the expression of genes related to inflammation have been seen as a mechanism and also as a potential target during I/R injury. Different studies have supported this view, as strategies such as ulinastatin administration to mice undergoing temporary middle cerebral artery occlusion, which downregulates TLR4 and NF-kB expression, sodium butyrate administered during I/R injury of the lung and inhibiting NF-κB and JAK2/STAT3 signaling pathways or combination of octreotide and melatonin to alleviate the inflammasome-induced pyroptosis through the inhibition of TLR4-NF-κB-NLRP3 pathway in liver I/R injury, have clearly showed that NF-kB plays an important role in reperfusion injury (Li et al., 2017b; El-Sisi et al., 2021; Ying et al., 2021).

Neutrophils can adhere to the endothelial wall where necrosis factors expressed by injured cells are exhibited on the luminal surface and contact the leukocytes (such as P-selectin). After flow reestablishment, the cells are able to cytoskeletal shape-shift and adapt to linear flow, moving through an inter-endothelial pattern and eventually localizing points of entry by mechanism of actin polymerization and matrix metalloproteinase activity and gaps between pericytes (Nourshargh and Alon, 2014). Other immune cells such as lymphocytes, thrombocytes, mast cells or macrophages are also believed to play a role in I/R injury by increasing the presence of tissue neutrophils (Rodrigues and Granger, 2010). Platelets are also involved in attracting leukocytes and inducing I/R damage by their activation in the presence of inflammatory cytokines including PAF, due to the damage of endothelial cells, lack of NO, prostacyclin, and abundance of ROS (Esch et al., 2010; Franks et al., 2010).

In response to brain hypoxia/ischemia, miRNAs modulate a complex network of gene expression, for which they were proposed as potential and reproducible biomarkers in ischemic stroke due to a consistent correlation with neuropathological changes and prognosis of stroke (Vijayan and Reddy, 2016; Condrat et al., 2020). Several types of hypoxia/ischemia-sensitive miRNAs, whose blood levels are correlated with their brain circulating levels, were identified as potential clinical biomarkers in stroke: miR-210, miR-125a-5p, miR-125b-5p, and miR-143-3p (Zeng et al., 2011; Tiedt et al., 2017). MiRNAs influence gene expression in response to hypoxic/ischemic injury, and in turn the inflammatory responses triggered by ischemia-hypoxia dysregulate miRNA expression (Chen et al., 2020b). In the complex array of neuroinflammatory events, microRNAs are at the center of target gene regulation and modulation, microglia activation, cytokine production, cell apoptosis, mitochondrial disfunction and immune cell development, maintaining the vicious processes that lead to the progression and extension of neuronal damage (Chen et al., 2020b).

**FIGURE 1:**
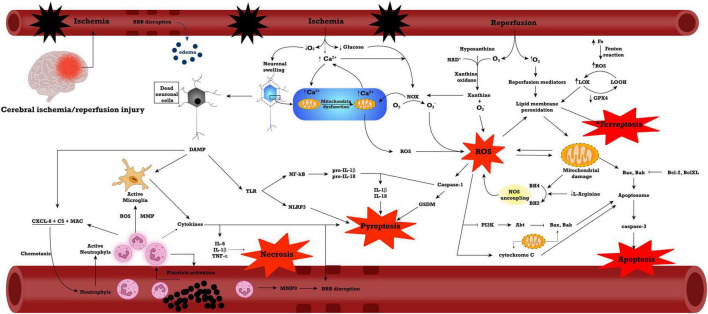
General mechanisms in ischemia/reperfusion injury. Abbreviations: Akt, Protein kinase B; BBB, blood-brain barrier; C5, complement fraction 5; CXCL, C-X-C Motif Chemokine Ligand; DAMP, damage-associated molecular pattern; GPX4, glutathione peroxidase 4; IL, interleukin; LOOH, lipid alcohol; LOX, lipid peroxide; MAC, membrane attack complex; MMP, matrix metalloproteinase; NAD, nicotinamide adenine dinucleotide; NF-kB, nuclear factor kappa-light-chain-enhancer of activated B cells; NOS, nitric oxide synthase; NOX, NADPH oxidase; PI3K, phosphoinositide 3-kinases; ROS, reactive oxygen species; TLR, Toll-like receptor; TNF-α, tumoral necrosis factor α.

The most important of these processes are displayed in Figure 1.

## microRNAs in Ischemia/Reperfusion Injury

### Inflammation

The inflammatory response is one of the major consequences of cerebral ischemia and miRNAs play an important role in its regulation. The involvement of several miRNAs in these pathways is presented in Tables 1, 2. Changes in the expression of inflammatory cytokines may occur after cerebral I/R injury (Wu et al., 2020). In lesions caused by I/R, inflammation is initiated by stagnant blood flow (vessel occlusion) and is then maintained by leukocytes activation and release of pro-inflammatory cytokines. Reducing or stopping the blood flow causes changes in the coagulation cascade, activates NF-kB and increases the expression of adhesion molecules on endothelial cells (Jurcau and Simion, 2021). Decreasing the amount of oxygen in the tissue causes varying degrees of damage. The first innate immune mechanism that is involved in this mechanism is the activation of toll-like receptors (TLRs). Activation of these receptors determines the activation of NF-kB, recognized as a pathway with a major role in the inflammatory response and with the ability to modulate several cytokines (TNF-α, IL-1β, and IL-6) and other mediators (iNOS, PGE2) (Shi et al., 2018; Yang et al., 2020). Microglia is the main factor involved in neuroinflammation. Its function and morphology are altered after ischemia. Activation of the microglia leads to its migration in and around the affected area (Hao et al., 2020). Together with the microglia, macrophages accumulate in the lesion (Islam et al., 2018). Following this activation process, the microglia release large amounts of pro-inflammatory cytokines (TNF-α, IL-6, IL-1β) that are considered to be the main factors involved in acute inflammation in ischemic stroke (Hao et al., 2020; Wang et al., 2020c).

**TABLE 1:** Up-regulated miRNAs in cerebral I/R injuries.

miRNA	miRNA or agmonir effect	Study type	References
miR-106b-5p	↑ ROS production, ↓antioxidant ability (SOD) and ↑ apoptosis activation	Experimental (Rat model and PC12 cell line)	Li et al., 2017a

miR-124	Biomarker of AIS	Clinical	Weng et al., 2011; Rainer et al., 2016

miR-124	↓ p-STAT3, ↓ pyroptosis	Experimental (Rat model)	Sun et al., 2020a

miR-125b	↓ CK2α; ↑ NOX2 and NOX4 activation, ↑ ROS	Experimental (Rat model and PC12 cell line)	Liang et al., 2018

miR-125b	↓ Protein kinase CK2	Experimental (PC-12 cell line)	Liang et al., 2018

miR-128	↓ proliferation ability ↓ GFAP and MAP2 ↑ TNF-α, IL-6, and IL-1β↓ GSH and SOD ↑ MDA ↓ ARPP21 and CREB1 ↓ BDNF	Clinical and Experimental (Mouse model and hippocampal neurons and astrocytes)	Chai et al., 2021

miR-128-3p	↓ Nrf2, ↓ antioxidant ability	Experimental (Rat model and neural stem cells line)	Li et al., 2019a

miR-142-5p	↓ Nrf2/ARE, ↑ ROS	Experimental (Rat model and primary hippocampal neurons)	Wang et al., 2017

miR-143-3p	↓ FSTL1, Bcl-2, ↑ Bax, caspase 3 and cleaved caspase 3, ↑ apoptosis	Experimental (Mice model and human neuroblastoma cell line SH-SY5Y)	Wang and Liu, 2021

miR-145	↑ ERK, p38 and MAPK ↑ Cyclin D1, Nestin, NSE, and GFAP ↓ Cleaved-caspase 3 ↑ NSCs proliferation ↑differentiation of NSCs ↓ apoptosis	Experimental (Rat model and Rat neural stem cells)	Xue et al., 2019

miR-150	↓ BBB permeability ↑ Tie-2 ↓ claudin-5	Experimental (Rat model and BMECs cell line)	Fang et al., 2016, 2

miR-153	↓ Nrf2 and HO-1, ↓ antioxidant levels, ↑ ROS	Experimental (Primary hippocampal neurons)	Ji et al., 2017

miR-16	Biomarker of AIS	Clinical	Rainer et al., 2016

miR-181a	↓ XIAP, Bcl-2, ↑ Bax, cleaved caspase 3, ↑ apoptosis	Experimental (Rat model and primary cortical neurons)	Zhang et al., 2019a

miR-182	↓ mTOR/ ↓ FOXO1 ↓ ZO-1, Occludin, and Claudin-5 ↓ Bcl-2/Bax	Experimental (Mouse model and primary cultures of astrocytes, mouse brain vascular pericytes, N2a mouse neuroblastoma cell line and BV2 microglial cells)	Zhang et al., 2020b

miR-187-3p	↓ Seipin, ↑ apoptosis, ↓ autophagy	Experimental (PC12 cells)	Ren et al., 2020

miR-191-5p	↓ BDNF	Experimental (Mouse model)	Wu et al., 2021

miR-195-5p and miR-451a	↓ BDNF ↓ VEGF-A	Clinical	Giordano et al., 2020

miR-19a-3p	↑ TNF-α, IL-1β, IL-6 ↓ Bcl-2, ↑ Bax ↓ IGFBP3 ↓ cell viability	Experimental (rat model and SH-SY5Y cell line)	Chai et al., 2020

miR-200a	↑ STAT and MAPK, ↑Bax/Bcl-2, p53, cytochrome c, ↑ apoptosis	Experimental (Neural stem cells)	Ma et al., 2017a

miR-200a-3p	↑ neuronal cell death, ↑ ROS levels	Experimental (HT-22 cells)	Wei et al., 2015

miR-200b-3p	↑ neuronal cell death, ↑ ROS levels	Experimental (HT-22 cells)	Wei et al., 2015

miR-20a	↑ Cadherin 1	Experimental (Rat model)	Yang et al., 2021

miR-210	↑HIF-1α, VEGF, caspase-3, ↑ apoptosis	Experimental (Rat model and rat neuronal cells)	Sun et al., 2019

miR-23a-3p	↓ NO, 3-NT ↑ MnSOD, ↑ antioxidant ability, ↓ caspase 3, ↓ ROS, ↓ apoptosis	*In vivo* and *in vitro* (Mice model and neuro-2a cells)	Zhao et al., 2014

miR-29b	↑ caspase 3, ↓ Bcl-2, MCL-1, ↑ apoptosis	Experimental (neuro-2a cells)	Huang et al., 2018

miR-302b-3p	↓ Nrf2/ARE, FGF15, ↑ caspase 3, ↑ ROS, ↑ apoptosis	Experimental (Murine HT22 cell line)	Zhang et al., 2019b

miR-30a	↑ BBB permeability ↑ zinc accumulation ↓ ZnT4 ↓ occludin and claudin-5	Experimental (Rat model and Brain microvascular EC bEnd3 cell line, pericyte cell line MBVP, astrocytic cell C8-D1A)	Wang et al., 2021c

miR-339	↓ FGF9 and CACNG2 ↓ Cell Proliferation ↑ Induces Apoptosis ↑ p-P38 and p-JNK	Experimental (PC12 cells)	Gao et al., 2020, 2

miR-421	↓ SOD, ↑ ROS, ↑ apoptosis	Experimental (Rat model and PC12 rat pheochromocytoma cell line)	Yue et al., 2020

miR-424	↑ Nrf2, ↑ antioxidant responses, ↓ROS	Experimental (Mouse model)	Liu et al., 2015

miR-429	↑ neuronal cell death, ↑ ROS levels	Experimental (HT-22 cells)	Wei et al., 2015

miR-670	↓ Hippo-Yap, ↑ apoptosis	Experimental (Mouse model and neuro-2a cells)	Yu et al., 2021c

miR-670	↓ phosphorylation of downstream Yap ↓ Yap degradation.	Experimental (Mouse model and neuro-2a cells)	Yu et al., 2020

miR-7-5p	↓ Sirtuin 1, ↑ apoptosis	Experimental (Rat model and SH-SY5Y cells)	Zhao and Wang, 2020

miR-9	Biomarker of AIS	Clinical	Ji et al., 2016

miR-93	↓ Nrf2 and HO-1, ↓ antioxidant levels, ↑ ROS	Experimental (mice model and primary cortical neurons)	Wang et al., 2016

*ACSL4, acyl-CoA synthetase long chain family member 4; AIM, absent in melanoma; AIS, acute ischemic stroke; Akt, Protein kinase B; ARE, antioxidant response element; AQP, Aquaporin; BBB, blood-brain barrier; BBC3, Bcl-2-binding component 3; BDNF, Brain-derived neurotrophic factor; CCL, C-C Motif Chemokine Ligand; CXCL, C-X-C motif ligand; CXCR, C-X-C motif chemokine receptor; FIP, FAK family-interacting protein; FOXO1, Forkhead box class O1; FSTL1, follistatin-like protein 1; GPX4, glutathione peroxidase 4; GSK, glycogen synthase kinase; HDAC, histone deacetylase; HIF, hypoxia inducible factor; HO-1, heme oxygenase 1; IGFBP3, Insulin Like Growth Factor Binding Protein 3; IL, interleukin; JAK, Janus kinase; MAPK, mitogen-activated protein kinase; MCL, myeloid leukemia sequence; MDA, malondialdehyde; miR, microRNA; MnSOD, manganese superoxide dismutase; mTOR, mammalian target of rapamycin; NEAT, nuclear paraspeckle assembly transcript; NF-κB, nuclear factor kappa-light-chain-enhancer of activated B cells; NO, nitric oxide; 3-NT, 3-nitrotyrosine; NOX, nicotinamide adenine dinucleotide phosphate (NADPH) oxidase; Nrf2, nuclear factor-erythroid factor 2-related factor 2; PI3K, Phosphoinositide 3-kinase; PUMA, p53-up-regulated modulator of apoptosis; SOD, super oxide dismutase; p-STAT, phosphorylated (activated) signal transducer and activator of transcription; RBFox-1, RNA-binding protein fox-1 homolog 1; SNAI2, Snail Family Transcriptional Repressor 2; SOX7, SRY-Box Transcription Factor 7; TFR1, transferrin receptor 1; TLR4, Toll-like receptor 4; TNF, tumoral necrosis factor; TP53INP1, Tumor Protein P53 Inducible Nuclear Protein 1; VEGF, vascular-epithelial growth factor; XIAP, X chromosome-linked inhibitor of apoptosis protein; ZO, zonula occludens.*

**TABLE 2:** Down-regulated miRNAs in cerebral I/R injuries.

miRNA	miRNA or agomiRNA effects	Study type	References
Let-7g* and miR-98	↓ CCL2, CCL5 (both miRNAs) ↓ CCL3, CXCL1 (Let-7g*) ↓ IP-10 (miR-98)	Experimental (Mouse model)	Bernstein and Rom, 2020

miR-124	↑ SOD, ↓ MDA and NOX2, ↓ NF-κB, TNF-α and IL-6, ↓ apoptosis	Experimental (Rat model and PC12 cell line)	Wu et al., 2020c

miR-125b	↓ p53, Bax, cytochrome C and caspase-3, ↓ apoptosis	Experimental (Rat model)	Xie et al., 2018

miR-126a-5p	↓ NOX2	Experimental (Rat model)	Liu et al., 2017

miR-130a	↑ PI3K/AKT	Experimental (Rat model, PC12 cells)	Zheng et al., 2019

miR-132-3p	↓ NOX4	Experimental (Rat model)	Liu et al., 2017

miR-132/212	↓ Claudin-1, TJAP-1, RBFox-1	Experimental (Mouse model and neuronal cultures)	Yan et al., 2021

miR-135b-5p	↓ GSK-3β activation, ↑ Nrf2/ARE, ↓ apoptosis	Experimental (Mouse hippocampal HT22 cell line)	Duan et al., 2018

miR-142-3p	↓ mitochondrial enzymes, ↑ mitochondrial function ↑ NOX2/Rac1, ↑ ROS, ↓ apoptosis	Experimental (Rat cerebrum primary cortical neurons)	Xia et al., 2020

miR-146a	↓ NOX4	Experimental (Rat model and SH-SY5Y cells)	Hong et al., 2018

miR-149-5	↓ S1PR2 ↓ pericyte migration ↑ N-cadherin ↑ BBB integrity	Experimental (Rat model and BMECs cell line and pericytes)	Wan et al., 2018

miR-150	↓ MYB ↓ VEGF	Experimental (BMVECs and 293T cells)	Zhang et al., 2021b

miR-182-5p	↓ TLR4	Experimental (Rat model)	Wang et al., 2018

miR-186	↓ HIF-1α ↓ N2a cell, cleaved caspase-3, Bax, ↑ Bcl-2 ↓ ROS production	Experimental (Rat model and Neuro2a cell line)	Li et al., 2021b

miR-18b	↓ Annexin A3, ↑ PI3K/Akt pathway, ↓TNF-α, IL-1β, ↓apoptosis	Experimental (Mouse model, SH-SY 5Y cells)	Min et al., 2020

miR-18b	↓ ANXA3 ↑ PI3K/Akt ↑ Bcl-2 ↓ Bax ↓ TNF-α, IL-1β ↑ p-PI3K, p-Akt, and p-mTOR	Experimental (Mouse model and SH-SY 5Y cell line)	Min et al., 2020

miR-194	↓ NOX1, ACSL4, Bach1, iron, ↑ GPX4, Nrf2, HO-1, ↓ ferroptosis	Experimental (PC12 cells)	Li et al., 2021d

miR–19a	↓ Syndecan 1, ↑ JAK1/STAT3 signalling pathway	Experimental (Mouse model)	Fang et al., 2021

miR-21	↓ MAPK	Experimental (Rat model)	Yao et al., 2018

miR-211	↓ PUMA, ↓ apoptosis	Experimental (Rat model and PC12 cells)	Liu et al., 2020

miR-214	↓ TFR1 and p53, ↑ GSH/GSSG, GPX4 ↓ ROS ↓ ferroptosis	Clinical and experimental study (Mouse model)	Lu et al., 2020a

miR-216a	↓ JAK2 ↓ p-STAT3 ↓ LDH ↓ cleaved caspase-3 ↓ iNOS, MMP-9, TNF-α, and IL-1b	Experimental (Mouse model and Primary Cortical Neuronal Cells)	Tian et al., 2018, 3

miR-219a-5p	↓ Phosphodiesterase 4D, ↓apoptosis,	Experimental (Mouse neuroblastoma N2a cells)	Lu et al., 2020b

miR-22	↓ NF-kB	Experimental (Rat model)	Yu et al., 2015

miR-22	↓ TNF-α, IL-1β, IL-6, IL-18, MIP-2 and PGE2 ↓ NF-κB ↓ p38 MAPK ↓ p-p38, NF-κB, COX-2 and iNOS	Experimental (Rat model and PC12 cells)	Dong et al., 2019

miR-22	↑ VEGF and Ang-1 ↑ p-PI3K/PI3K and p-Akt/Akt	Experimental (Rat model and	Wang et al., 2020b

miR-22-3p	↓ IL-1β, IL-18, ↓ cleaved caspase 1 ↓ NLRP3, NEAT1, ↓pyroptosis, ↓ apoptosis	Experimental (Rat model and rat primary cortical neurons)	Zhang et al., 2021a

miR-224-3p	↓ FIP200, ↓ cleaved caspase-3, ↓ ROS, ↓ apoptosis	Experimental (Neuro-2a cells)	Deng et al., 2019

miR-25	↓ Fas/FasL, ↓ inhibits apoptosis	Experimental (Human SH-SY5Y and IMR-32 cells)	Zhang et al., 2016

miR-25	↓ NOX4	Experimental (Rat model and SH-SY5Y cells)	Hong et al., 2018

miR-27a-3p	↓ FOXO1, ↓ caspase 3, caspase 9, ↑ Bcl-2, ↑ SOD, GSH, ↓ MDA, ↓ apoptosis, ↓ ROS	Experimental (Rat model and murine HT22 cells)	Li et al., 2021c

miR-27a-3p	↓ BBB permeability ↑ claudin-5 and ↑ occludin, ↓ GSK3ß ↑ Wnt/ß-catenin.	Experimental (hCMEC/D3 cell line)	Harati et al., 2022

miR-29a	↓ BBC3/PUMA, ↓ apoptosis	Experimental (Mouse primary astrocyte cells)	Ouyang et al., 2013

miR-29a-5p	↓ NOX4	Experimental (Rat model)	Liu et al., 2017

miR-29c-3p	↓ NOX4	Experimental (Rat model)	Liu et al., 2017

miR-29b	↓ AQP-4 ↓ Extravasated IgG ↑ CD31/occludin and CD31/ZO-1	Clinical and experimental (Mouse model)	Wang et al., 2015b, 4

miR-320a	↓ AQP-1 and AQP-4	Experimental (Rat model and Human astrocytoma cells)	Sepramaniam et al., 2010

miR-326-5p	↓ STAT3, ↑ Mitofusin 2	Experimental (Rat model)	Huang et al., 2021b

miR-34b	↓ Keap1, ↑Nrf2/ARE, HO-1, ↓ NO, 3-NT, ↑ SOD, MnSOD, ↓ ROS	Experimental (Rat model and cell line)	Huang et al., 2019

miR-34c-5p	↑ Bcl-2, ↓ p65, Bax/β-actin, caspase-3, ↓ IL-6, TNF-α, ↑ IL-10, ↓ apoptosis	Experimental (Rat model and cortical neurons)	Tu and Hu, 2021

miR-34c-5p	↓ p65, NF-kB, ↓ Nuclear Receptor Coactivator 1	Experimental (Rat model)	Tu and Hu, 2021

miR-374	↑ Wnt5a, Bcl-2, Bcl-Xl, ↓ Bax, ↓ apoptosis	Experimental (Rat model)	Xing et al., 2021

miR-374	↓ Wnt5a ↓ BAX ↑ BCL-XL and BCL-2	Experimental (rat model)	Xing et al., 2021

miR-376b-5p	↑ Wnt3a and β-catenin ↓ SOX7 ↓ BBB permeability	Experimental (Mouse model)	Zhao et al., 2021

miR-410	↓ TIMP2 ↓ ERK, ↓ p38 MAPK, ↓ JNK, ↓ p-ERK, and p-JNK ↓ MDA ↑ SOD, GSH-Px	Experimental (Mouse model and culture of hippocampal neurons)	Liu et al., 2018

miR-424	↑ SOD, MnSOD, Nrf2 ↓ MDA, ↓ ROS ↓ apoptosis Increased antioxidant ability (SOD and Nrf2) and decreased ROS and MDA	Experimental (Mice model and primary cortical neurons)	Liu et al., 2015

miR-484	↓ BCL2L13, ↓ apoptosis	Experimental (Mouse model and murine cortical neurons)	Liu et al., 2021

miR-485	↓ AIM2, caspase 1, ↓ IL-1β, IL-18 ↓ apoptosis and pyroptosis	Experimental (Rat model and human neuroblastoma cells)	Liang et al., 2020

miR-489-3p	↓ HDAC2, ↓ apoptosis	Experimental (Rat model and PC12 cells)	Jia et al., 2022

miR-496	↓ BCL-2-like protein 14, ↓ apoptosis	Experimental (Rat model and SH-SY5Y cells)	Yao et al., 2019

miR-532-3p	↓ NOX2, caspase 3 ↓ ROS, ↓ apoptosis	Experimental (Rat model and SH-SY5Y cells)	Mao et al., 2020

miR-532-5p	↓CXCL1/CXCR2/Nf-kB, ↓ apoptosis	Experimental (Rat model and SH-SY5Y cells)	Shi et al., 2021

miR-539	↓ Matrix metallopeptidase 9 ↓ SNAI2	Clinical and Experimental (rat model + RBMVEC cell line)	Li et al., 2021a, 9

miR-652	↓ NOX2, ↓ ROS	Experimental (Rat model and SH-SY5Y cells)	Zuo et al., 2020

miR-7-5p	↓p65, TNF-α, IL-6, IL-1, ↓ROS, ↓ apoptosis	Experimental (Rat model and PC12 cells)	Xu et al., 2019

miR-7a-5p	↓α-synuclein, ↓ apoptosis	Experimental (Rat model)	Kim et al., 2018

miR-874-3p	↓ Bcl2 Modifying Factor and BCL2 Like 13	Experimental (Rat model, SH-SY5Y cells)	Jiang et al., 2019

miR-92a	↓ NOX4	Experimental (Rat model and SH-SY5Y cells)	Hong et al., 2018

miR-92b	↓ BBB permeability ↑ claudin-5 ↑ occluding, ZO- 1 and VE- cadherin ↑ SOD ↓ ROS ↓ NOX4	Experimental (rat model and BMECs cel line)	Shen et al., 2021, 4

miR-98	↓ leukocyte infiltration and ↓ microglia activation	Experimental (Mouse model and primary BMVEC)	Bernstein et al., 2020

miR-98-5p	↓ Nrf2/ARE, ↑ Bach1, ↓ ROS, ↓ apoptosis	Experimental (murine hippocampal neuronal cells)	Sun et al., 2018b

miR-98-5p	↑ SOD, Bcl-2, HO-1 ↓ Bax2, cleaved caspase 3 ↓ROS, ↓ apoptosis	Experimental (Mouse model)	Yu et al., 2021b

miR-99a	blocks aberrant S phase re-entry, ↓ caspase-3/β-actin ↓apoptosis	Clinical and experimental (Patients, mouse model, neuro-2a cells)	Tao et al., 2015

*ACSL4, acyl-CoA synthetase long chain family member 4; AIM, absent in melanoma; AIS, acute ischemic stroke; Akt, Protein kinase B; ARE, antioxidant response element; AQP, Aquaporin; BBB, blood-brain barrier; BBC3, Bcl-2-binding component 3; BDNF, Brain-derived neurotrophic factor; CCL, C-C Motif Chemokine Ligand; CXCL, C-X-C motif ligand; CXCR, C-X-C motif chemokine receptor; FIP, FAK family-interacting protein; FOXO1, Forkhead box class O1; FSTL1, follistatin-like protein 1; GPX4, glutathione peroxidase 4; GSK, glycogen synthase kinase; HDAC, histone deacetylase; HIF, hypoxia inducible factor; HO-1, heme oxygenase 1; IGFBP3, Insulin Like Growth Factor Binding Protein 3; IL, interleukin; JAK, Janus kinase; MAPK, mitogen-activated protein kinase; MCL, myeloid leukemia sequence; MDA, malondialdehyde; miR, microRNA; MnSOD, manganese superoxide dismutase; mTOR, mammalian target of rapamycin; NEAT, nuclear paraspeckle assembly transcript; NF-κB, nuclear factor kappa-light-chain-enhancer of activated B cells; NO, nitric oxide; 3-NT, 3-nitrotyrosine; NOX, nicotinamide adenine dinucleotide phosphate (NADPH) oxidase; Nrf2, nuclear factor-erythroid factor 2-related factor 2; PI3K, Phosphoinositide 3-kinase; PUMA, p53-up-regulated modulator of apoptosis; SOD, super oxide dismutase; p-STAT, phosphorylated (activated) signal transducer and activator of transcription; RBFox-1, RNA-binding protein fox-1 homolog 1; SNAI2, Snail Family Transcriptional Repressor 2; SOX7, SRY-Box Transcription Factor 7; TFR1, transferrin receptor 1; TLR4, Toll-like receptor 4; TNF, tumoral necrosis factor; TP53INP1, Tumor Protein P53 Inducible Nuclear Protein 1; VEGF, vascular-epithelial growth factor; XIAP, X chromosome-linked inhibitor of apoptosis protein; ZO, zonula occludens.*

I/R damage can be ameliorated by transforming growth factor β1 (TGF-β1), a cytokine with anti-inflammatory effects (Yang et al., 2020). TGF-β1 is a factor produced in large amounts in the lesion, starting on day 5 after reperfusion or later. A source of TGF-β1 may be the microglia and macrophages. The anti-inflammatory effect of TGF-β1 is thought to be a consequence of phosphorylation of the Smad protein by binding of this ligand to TGF-β receptors (Islam et al., 2018). Another member of the TGF family, TGF-β2, has a neuroprotective effect, being considered a neuroprotective factor. The expression of this protein is increased in animals with transient cerebral ischemia. Activation of the TGF-β2/Smad3 signaling pathway is essential for neuroprotection in ischemic brain injury (Peng et al., 2019).

The inflammatory response can be initiated by inflammasomes, complex molecular protein structures that are sensitive to cellular changes when homeostasis is lost (Franke et al., 2021). The main components of an inflammasome are a NLR sensor molecule, a pro-inflammatory caspase, and an adaptor protein (apoptosis-associated speck-like protein (ASC)) with a role in transmitting cellular signals (Hong et al., 2019; Caseley et al., 2020). Currently, the most studied inflammasome is nod-like receptor protein 3 (NLRP3). It plays an important role in various diseases with inflammatory components. Activation of NLRP3 leads to cerebral ischemia by releasing proinflammatory cytokines, such as IL-1β and IL-18. In the first stage after cerebral I/R injury, microglia become the main reservoir for activated NLRP3 inflammasome. In the following stages, NLRP3 are activated in both neurons and endothelial cells (Gao et al., 2017; Gong et al., 2018). The interaction between inflammasomes and TXNIP (thioredoxin interacting protein) leads to the activation of inflammation. In a normal, stress-free state, TXNIP is linked to Trx1 (thioredoxin1). Thus, NLRP3 is in inactive form. In stroke, a state with high oxidative stress, TXNIP and Trx1 dissociate and thus NLRP3 is activated. Nuclear factor erythroid 2-related factor 2 (Nrf2) is involved in the oxidative process and can interfere with processes that are consequences of oxidative stress. Trx1 has a neuroprotective effect against I/R and Nrf2 lesions by regulating the Trx1/TXNIP interaction negatively regulates NLRP3 inflammasome (Hou et al., 2018).

### Cell Death

In I/R injuries, the first pathological event is represented by hypoxia due to ischemia. This causes cell death by mitochondrial damage and ROS formation. In the following phases, several inflammatory pathways are activated, besides the initial ROS events, all of which contribute to neuronal damage and loss of function (Jurcau and Simion, 2021).

#### Necrosis and Necroptosis

Necrosis is the main form of cell death present in the hypoxic regions closest to the ischemic core. It is characterized by plasma membrane permeation and cell and organelle swelling (D’Arcy, 2019). It is caused by the intense stress caused by the lack of oxygen and nutrients in the ischemic areas. Necroptosis shares similar death-pattern characteristics to necrosis, but it is controlled by death signals and therefore, it is considered a form of programmed cell death (Wu et al., 2018). Necroptosis requires the presence of death signals, such as tumoral necrosis factor (TNF) receptor and the activity of receptor-interacting protein 1 (RIP1 or RIPK1) (Festjens et al., 2007; Vandenabeele et al., 2010). In cerebral I/R injuries, inhibiting RIP1 reduces the neuronal damage (Degterev et al., 2008; Kim et al., 2017a). Several other therapeutic approaches have been tested in murine models for reducing necroptosis, however, the data regarding miRNAs is scarce (Liao et al., 2020). Among the studies miRNAs, miR-497 and miR-369 seem to have a role in necroptosis by influencing the cellular response to TNF-α (Hsu et al., 2020; Yin et al., 2022).

#### Apoptosis

Compared to necrosis, apoptosis is a coordinated formed of programmed cell death. It involves the activation of a complex cascade of processes and the activation of caspases, cysteine proteases with a pivotal role in this process (Elmore, 2007). In I/R injuries, it is present both in the initial hypoxic phase, as well as in the reperfusion state, but activated *via* different pathways (Wu et al., 2018). In the hypoxic phase, the intrinsic pathway plays a more important role, caused by the hypoxia-induces mitochondrial damage, which leads to the formation of apoptosomes and the activation of caspase 9, which leads to the activation of caspase 3 and the execution pathway. In the reperfusion state, the inflammatory mediators present in large amounts are responsible for the activation of the extrinsic pathway, where caspase 8 activation leads to caspase 3 activation and the execution pathway that includes DNA degradation, cytoskeletal reorganization and in the end, the formation of apoptotic bodies and cell death (Radak et al., 2017).

Apoptosis inhibition strategies were found to be effective in cerebral ischemia-reperfusion injury models, by reducing the extent of the infarct volume and improving the neurological score (Gong et al., 2017; Tang et al., 2020; Wang et al., 2021a). Biochanin A, an O-methylated isoflavone, reduced the expression of pro-apoptotic proteins Bax, Bcl-2, caspase-3 and caspase-12 in a model of middle cerebral artery occlusion and reperfusion (MCAO) (Guo et al., 2021b). Also, astragalin, another flavonoid reduced the expression of Bax and caspase-3, while upregulating the expression of Bcl-Xl (Chen et al., 2020a). Among these strategies, miRNA-based therapeutic approaches are presenting promising experimental results (Sun et al., 2018a; Liu et al., 2019b).

One of the most studied miRNAs in I/R pathologies is miR-124 (Liu et al., 2019b). In a rat model of MCAO, miR-124 presented as a promising biomarker for cerebral stroke injuries (Weng et al., 2011). Also, in patients with ischemic stroke, miR-124 as well as miR-9 were significantly elevated, supporting the idea of using miRNAs as biomarkers in I/R injuries (Ji et al., 2016). Another study in stroke patients showed the utility of miR-124-3p and miR-16 as biomarkers (Rainer et al., 2016).

In an experimental study, miR-211 downregulation increased the neurological damage and infarct volume of the mouse brain *via* a loss of Bcl-2-binding component 3 (BBC3) inhibition (Liu et al., 2020). BBC3 is also known as p53-up-regulated modulator of apoptosis and is part of the Bcl-2 protein family. Its main mechanism of action is interacting with other Bcl-2 family members proteins and promoting apoptosis (Nakano and Vousden, 2001). By upregulating miR-211, BBC3 was inhibited and the infarct size, neurological score and apoptosis were decreased. Another miRNA that acts by inhibiting BBC3 is miR-29a. In transient forebrain ischemia, miR-29a levels were decreased in the ischemic areas and its upregulation provided a protective effect in I/R injury (Ouyang et al., 2013). MiR-7-5p was upregulated in I/R injury models, degrading Sirtuin 1, a protein which alleviates I/R injuries, and therefore increasing neuronal apoptosis (Zhao and Wang, 2020; Diwan et al., 2021). In another study, miR-7-5p expression was reduced in MCAO rat models and its increase reduced the formation of ROS and inflammatory molecules and reduced the associated neuronal apoptosis (Xu et al., 2019). Similar results were found by Kim et al. in a rat model of I/R, where miR-7-5p levels were downregulated and pre-ischemic administration of miR-7 reduced I/R associated apoptosis and neuronal injury (Kim et al., 2018). The regulation of several other miRNAs has been studied in correlation with pro-apoptotic proteins or apoptosis, which are presented in Tables 1, 2.

#### Pyroptosis

Pyroptosis is considered a gasdermin (GSDM)-mediated programmed cell death (Shi et al., 2015). Compared to apoptosis, pyroptosis includes in its characteristics inflammation, as well as pore formation and cell swelling, with loss of cell membrane integrity. It includes the activation of caspases, however, these are different than in apoptosis, pyroptosis being activated by caspases 1, 4, 5, and 11 (Yu et al., 2021a). The canonical pathway in pyroptosis is characterized by cleaved-caspase 1 inflammasome formation, GSDM cleavage and release of IL-1β and IL-18 (Nunes and de Souza, 2013). The process by which pyroptosis is activated has been reviewed in detail by Yu et al. (2021a).

In cerebral I/R injuries, pyroptosis inhibition through the NF-kB pathway reduced the infarct volume and improved the neurological recovery. Also, inhibition of inflammasome formation *via* NLRP3 and NLRP1 regulation proved successful in improving neuronal survival and diminishing the impact of I/R injuries (Chen et al., 2020a; Sun et al., 2020b; Huang et al., 2021a). In this process, several miRNAs have been profiled to be activated and possible therapeutical targets for pyroptosis inhibition (Wang et al., 2020a). Gastrodin regulated the miR-22/NEAT1 axis and reduced the pro-inflammatory cytokines, reducing pyroptosis and attenuating the I/R injuries both *in vivo* and *in vitro* (Zhang et al., 2021a). MiR-124, which was previously discussed for apoptosis and was described as a marker of I/R injury, inhibits STAT3 expression and thereby reduces pyroptosis and improves the neurological outcome (Sun et al., 2020a). Overall, more studies are needed in order to fully elucidate how miRNAs regulation is related to pyroptosis and how these could potentially be used as therapeutic targets.

#### Ferroptosis

Ferroptosis is a recently described form of iron dependent cell death (Zhang et al., 2021c). Intracellular iron accumulation leads through the Fenton reaction to the formation of hydroxyl radicals that are ROS. ROS formation leads to lipid peroxidation (mainly phosphatidylethanolamine polyunsaturated fatty acids) that are destroying the lipid membranes, causing cell death. Ferroptosis is involved in several pathologies, including inflammatory pathologies, neurodegenerative diseases, cancers and I/R injuries (Liang et al., 2019; Capelletti et al., 2020; Li et al., 2020; Reichert et al., 2020; Sun et al., 2020c; Mitre et al., 2022). In mice experimental models of I/R injury, ferroptosis inhibition reduces the intestinal ischemic area and also protects the lungs and liver against ischemia-induced remote injuries (Li et al., 2019b,^2020^; Qiang et al., 2020; Deng et al., 2021). In acute myocardial infarction, ferroptosis inhibition by liproxstatin-1 presented promising results by reducing the infarct size in experimental studies (Lillo-Moya et al., 2021). More studies are needed to determine the clinical efficiency of ferroptosis-inhibiting strategies in I/R injuries.

In cerebral I/R injury, tau-mediated iron accumulation can trigger ferroptosis (Tuo et al., 2017). Ferroptosis activation increases the neuronal damage and the ischemic area (Zhao et al., 2022). Inhibiting this process by enhancing the expression of GPX4, the main regulatory enzyme of ferroptosis, leads to reduced neuronal deficit after ischemia and reduced neuronal death (Guan et al., 2019, 2021). These results are similar with other experimental studies, where ferroptosis inhibition by inhibiting its various pathways improved the neurological outcome and reduced the affected area in I/R injuries (Chen et al., 2021; Guo et al., 2021a; Wang et al., 2021b; Tuo et al., 2022; Xu et al., 2022).

In patients with acute ischemic stroke, miR-214 levels were downregulated. In mice, upregulating the levels of miR-214 reduced the infarct size and improved the neurological scores (Lu et al., 2020a). In oxygen-glucose deprivation, miR-194 upregulation improved cell survival and viability, as well as reduced the expression of ACSL4, while upregulating GPX4. These results indicate that miR-194 could potentially reduce ferroptosis and thus improve neuronal survival *in vivo* (Li et al., 2021d).

### Oxidative Stress Damage

#### The Role of Oxidative Stress in Cerebral Ischemia-Reperfusion Injury (CIRI)

In I/R injuries, the reperfusion process provides a large amount of oxygen carried by the red blood cells to the ischemic site. At the same time, the rapid alterations in oxygen flow allows the generation of ROS. Ischemia also modifies the concentration of antioxidative agents, which leads to greater damage caused by the generated ROS. In the ischemia stage, ATP production is reduced. Consecutively, the function of ion-exchange channels and enzymes is altered, leading to mitochondrial dysfunction and electrolytes imbalance. In these circumstances, the oxidative stress pathways are further activated: the NADPH oxidase (NOX) complex, the inducible nitric oxide (iNOS) complex and the xanthine oxidase complex (Wu et al., 2018).

Mitochondria is the main source for ROS synthesis due to the electron chains from the mitochondrial inner membrane, NOXs and mitochondrial redox carriers complexes I and III. In physiological states, the generation of ROS, like superoxide anion, hydrogen peroxide and hydroxide radical, is at a low level and antioxidants, like superoxide dismutase (SOD), catalases, glutathione peroxidase (GSHPx) and glutathione, control any excess of ROS (Hu et al., 2015). The excessive production or delayed elimination of ROS is often a starting point for CIRI. An excessive amount of ROS in the brain interacts with structural molecules, such as proteins, lipids, carbohydrates and nucleic acids, affecting the neuronal biochemical processes and promoting neuronal death. The main mechanisms involved in ROS toxicity are: mitochondrial membrane lipid peroxidation, cross-linking of molecules, like nucleic acids, proteins and carbohydrates that alter their function in biochemical processes, endothelial damage of the BBB and consecutively increased permeability, activation of inflammatory key factors, like cytokines and adhesion molecules, and increased synthesis of excitatory amino acids (EAA), involved in delayed neuron death (Wu et al., 2020).

#### Oxidative Stress

Oxidative stress is involved in DNA damage, local inflammation and endothelial dysfunction. Nuclear factor (erythroid-derived 2) -related factor 2 (Nrf2) is an antioxidant regulator activated in oxidative stress conditions that upregulate the expression of antioxidant genes, like superoxide dismutase (SOD), heme-oxygenase-1 (HO-1), NADPH- quinone oxidoreductase 1 (NQO1) and glutathione S transferase (GST) (Chen et al., 2015).

Li et al. (2019a) showed that theaflavin has an antioxidant and neuroprotective effect in a rat model of I/R injury and in neural stem cells subjected to oxygen-glucose deprivation and reoxygenation (OGD/R), increasing the expression of Nrf2 by downregulating miRNA-128-3p. The study confirmed that the miRNA-128-3p level of expression is increased in CIRI, and it is responsible for ROS generation.

Zhao et al. (2014) demonstrated that miR-23a-3p is increased in a CIRI mice model, a protective trial mechanism activated to increase the antioxidant ability of the neurons and to suppress oxidative stress. MiR-23a-3p agomir decreased the synthesis of nitric oxide (NO), 3-nitrotyrosine and hydrogen peroxide-induced lactate dehydrogenase release and increased the expression of manganese superoxide dismutase, an enzyme that protects the mitochondrial energy network from oxidative stress damage. Another similar study found out that miR-424 levels increased at 1 and 4 h and decreased at 24 h after reperfusion in an I/R mice model. MiR-424 agomir decreased the level of excessive ROS and lipid peroxidation product malondialdehyde (MDA) generated after reperfusion and increased the expression of SOD and Nrf2. The study concluded that miR-424 activates an antioxidant mechanism in CIRI to limit further damage (Liu et al., 2015).

Huang R and the collaborators suggested that the reduced level of miR-34b expression in focal cerebral I/R is associated with oxidative stress parameters and decreased antioxidant ability. They showed that overexpression of miR-34b ameliorates CIRI through suppression of Keap1 and increase of Nrf2 and heme oxygenase (HO-1). Kelch-like ECH-associated protein 1 (Keap1)/Nrf2/ARE signaling pathway has been proved to be an important antioxidant mechanism and a potential target for miR-34b (Huang et al., 2019). Nrf2/ARE inhibition and excessive ROS production are common mechanisms that involve other miRNAs downregulation, such as miR-98-5p or miR-135b-5p (Duan et al., 2018; Sun et al., 2018b).

Wei et al. (2015) concluded that the miR-200 family increases ROS production, reduces mitochondrial membrane potential and modulates apoptosis network during CIRI, especially miR-200a-3p, miR-200b-3p and miR-429. The imbalance between ROS excessive production (MDA) and reduced antioxidant (SOD) ability causing oxidative stress damage is also determined by miR-106b-5p upregulation. MiR-106b-5p accentuates neurons death by involving the Bcl-2 family proteins, with the pro-apoptotic protein Bax and antiapoptotic protein B cell lymphoma-2 balance dysregulation (Bcl-2). Li et al. (2017a) reported that miR-106b-5p antagomir ameliorates the oxidative stress imbalance and activates antiapoptotic proteins, like Bcl-2 and myeloid cell leukemia-1 (Mcl-1). MiR-421 is also upregulated in CIRI and seems to activate the same pathological mechanisms (Yue et al., 2020). Nrf2/ARE mediated antioxidant pathways inhibition and ROS excessive production were described in a large number of studies referring to miRNAs upregulation: miR-153 (Ji et al., 2017), miR-93 (Wang et al., 2016), miR-142-5p (Wang et al., 2017) and miR-302b-3p that also targets fibroblast growth factor 15 (FGF15) (Zhang et al., 2019b).

#### Mitochondria Damage

Mitochondrial pathways involved in the survival of the cell are ATP production and synthesis of different molecules used in signaling networks. Mitochondria environment is also a place for miRNAs mediated posttranscriptional regulation, affecting energy metabolism, biochemical homeostasis and the activity of enzymes related to oxidative stress pathways. In CIRI, mitochondrial damage is involved in pathophysiological processes, such as ROS excessive production, reduced antioxidant activity, energy metabolism dysregulation and neuronal apoptosis (Hu et al., 2015).

To establish a possible interaction between miRNAs and mitochondrial damage, Xia et al. (2020) designed a model of OGD/R in primary cortical neuron culture. They proved that the decreased expression of miR-142-3p is involved in mitochondrial dysfunction and suggested that miR-142-3p regulates enzymes involved in mitochondrial biogenesis and function, such as electron transfer chain complexes I-III, peroxisome proliferator-activated receptor- γ coactivator-1α (PGC1α), mitochondrial transcription factor A (TFAM), and nuclear respiratory factor 1 (NRF1). Moreover, miR-142-3p overexpression improves mitochondrial function by decreasing the ROS toxic effects due to inhibition of NOX2/Rac Family Small GTPase 1 (Rac1)/ROS signaling pathway (Xia et al., 2020).

#### NADPH, iNOS

NADPH oxidase (NOX) is a family of 7 enzymes, NOX1 to NOX5 and dual oxidase (Duox-1 and Duox-2). NOX2 and NOX4 have been described as important enzymes that coordinate neuronal apoptosis and ROS generation in CIRI (Liang et al., 2018; Zuo et al., 2020).

Protein kinase CK2 (casein kinase 2) is a kinase that phosphorylates a large number of different substrates; therefore, it is involved in different cellular processes. It has been outlined that CK2 has a neuroprotective effect in CIRI by downregulating NADPH oxidases NOX2 and NOX4. Both *in vivo* and *in vitro* studies concluded that miR-125b is upregulated in I/R injury, while CK2α is decreased and proved that mi-R-125b binds with 3′UTR of CK2α and directly suppresses CK2 levels, resulting in NOX2 and NOX4 activation and ROS overproduction and neuronal apoptosis (Liang et al., 2018). Zuo et al. (2020) showed that miR-652 is significantly decreased, while the expression of NOX2 is increased in a CIRI rat model and in a cell hypoxia/reoxygenation (H/R) model. Overexpression of miR-652 in H/R cells reduced NOX2 expression and ROS production and ameliorated brain tissue CIRI (Zuo et al., 2020). A similar study that used both *in vitro* and *in vivo* CIRI models found out that miR-532-3p level of expression is reduced and NOX2 level is increased and suggested that miR-532-3p downregulation may be a part of CIRI through the NOX2 pathway (Mao et al., 2020).

The downregulation of several miRNAs in the ischemic brain tissue in hyperglycemic rats has been associated with NOX2 and NOX4 genes: miRNA-29a-5p, miRNA-29c-3p, miRNA-126a-5p, miRNA-132-3p, miRNA-136-3p, miRNA-138-5p, miRNA-139-5p, miRNA-153-5p, miRNA-337-3p, and miRNA-376a-5p. NOX2 was identified as the target gene of miR-126a-5p whereas NOX4 was the target gene of miR-29a-5p, miR-29c-3p and miR-132-3p (Liu et al., 2017). NOX4 was also studied as a target for miR-25, miR-92a and miR-146a. In an experimental study of CIRI, the expression levels of miR-25, miR-92a and miR-146a were decreased, but the NOX4 protein expression was increased in the interventional group. Treatment with isoflavones resulted in decreased ROS generation and neuronal cell death related to the inhibition of NOX4 *via* the induction of NOX4-related miRNAs (Hong et al., 2018).

### Other Pathways

#### Blood Brain Barrier Disruption

Alongside with oxidative stress, apoptosis and inflammation, disruption of BBB and subsequent increased permeability of BBB, results in myelin sheath damage and brain edema, leading to neuronal dysfunction (Haley and Lawrence, 2017; Jiang et al., 2018a; Ma et al., 2020). BBB dysfunction has been ascertained in multiple brain disorders, including stroke, traumatic brain injury (TBI), MS, epilepsy, AD, amyotrophic lateral sclerosis and PD (Daneman, 2012; Kamphuis et al., 2015). The main pathways activated upon BBB disruption consists of tight junction protein degradation, microvascular endothelial cells (ECs) damage, immune cell infiltration and activation of cytokine expression (Shen and Ma, 2020). MiRNAs have been shown to modulate BBB function under various pathological conditions, from: ischemic brain injury, TBI, spinal cord injury to neurodegenerative diseases (AD, Vascular dementia), brain tumors and cerebral infections (Ma et al., 2020).

In MCAO-induced CRTC1 knockout mice model, reduced levels of miRNA-132/212 have been correlated with aggravated BBB permeability and increased infarct volume. Moreover, miRNA-132 promotes BBB integrity expression, by binding to 3-UTR regions of the target genes of tight junction-associated protein-1 (TJAP-1), claudin-1, thus repressing junction protein’s expression (Yan et al., 2021). Peripheral blood samples of 48 cerebrovascular patients revealed decreased levels of miR-539, which was related to impaired BBB. By binding to SNAI2, miR-539 has been shown to restore endothelial cell permeability by repressing MMP9 signaling pathway (Li et al., 2021a).

The expression of intercellular junctions could also be regulated by miR-27a-3p mimics *via* upregulating the protein expression of claudin-5 and occludin, thus impairing BBB permeability in CMEC/D3cells model (Harati et al., 2022). In MCAO-induced miR-182 KD (knockout) mice, the integrity of BBB was restored, with increased expression of tight junction proteins (Zhang et al., 2020b).

The cellular components of BBB have also been regulated by miRNAs upon ischemic insult. In ischemic rat brain and cultured pericytes, miR-149-5 expression was decreased. Downregulation of miR-149-5p expression enhances S1PR2 in pericytes, which was associated with decreased N-cadherin expression and increased pericyte migration, thus aggravating BBB integrity. Intracerebroventricular injection of agomir-149-5p has been shown to increase the level of N-cadherin and decrease pericyte migration, ameliorating BBB dysfunction (Wan et al., 2018).

Vascular endothelium poses important roles in BBB homeostasis and integrity (Hawkins and Davis, 2005). The integrity of BBB depends on the ‘injury’ status of brain microvascular endothelial cells (BMECs), suggesting that protecting BMECs represents a therapeutic strategy against ischemic stroke. CI/R injury induces autophagy in BMECs, and in turn autophagy further protects BMECs upon CI/R injury, suggesting the protective mechanism of autophagy on BMECs exposed to OGD/R injury (Li et al., 2014a). Ln RNA Malat1 promotes down-regulation of miR-26b to promote neuroprotective effects in CI/R injury by stimulating autophagy of BMECs (Li et al., 2017c).

#### JAK2, STAT3, MAPK Associated Pathways

Multiple studies evidenced that JAK2/STAT3 signaling pathways have been activated after ischemic stroke, posing neuropathogenic roles in I/R injury (Liang et al., 2016). Interestingly, silencing JAK2/STAT3 pathway has been associated with up-regulation expression levels of miRNAs in various pathological settings, including hepatopulmonary syndrome rat model, pancreatic cancer cells (Wang et al., 2015a; Yin et al., 2022).

In MCAO mice model and OGD-induced neuronal cells dysfunction, miR-216a was down-regulated. Overexpression of miR-216a exhibited neuroprotective effects against I/R injury by negatively regulating JAK2/STAT3 signaling pathway (Tian et al., 2018).

Mitogen-activated protein kinases pathway (MAPKs) participate in signal transduction, exerting regulatory roles on cell death and survival, being involved in different biological processes, including differentiation, cell proliferation and apoptosis (Nozaki et al., 2001; Imajo et al., 2006). Under ischemic conditions, MAPK activated inflammatory processes and promoted neuronal cell death, the expression level of MAPK being highly expressed in the cerebral macrophages from the ischemic core after stroke (Madhyastha et al., 2012; Wang et al., 2019; Xie et al., 2019; Zeng et al., 2019).

MiR-22 ameliorates the neuroinflammatory responses *in vivo* and *in vitro* animal models of I/R injury, by suppressing p38 MAPK/NF-κB pathways (Dong et al., 2019). In ischemic rat model, miR-145 exhibited low expression levels, which was associated with suppressing the MAPK pathways. Interestingly, in rat neuronal stem cells (NSCs), miR-145, p38 and ERK increased in a cultured time-dependent manner, suggesting the neuroprotective mechanisms promoted with growth of the NSCs. miR-145 promoted NSCs proliferation and inhibited apoptosis, whereas MAPK’s inhibitor (SB203580) enhanced apoptosis and inhibited NSCs proliferation. After cerebral injection of NSCs in the ischemic rat cortex, the walking ability and neurological impairment of ischemic stroke rats improved over time, miR-145 playing critical roles in NSCs-promoted recovery of ischemic rat cortex, by targeting MAPK pathway (Xue et al., 2019). Moreover, miR-339 accelerated the progression of I/R injury in MCAO-rat model and PC12 cells exposed to OGD/R treatment, by stimulating proliferation and apoptosis of neuronal cells. The deleterious effects of miR-339 on neuronal injury proceed *via* inhibiting FGF9/CACNG2 axis, thus activating MAPK signaling pathway in ischemic stroke (Gao et al., 2020, 2). MiR-410 exhibited low levels in I/R mouse model and miR-410 mimic transfection reversed neuron apoptosis and enhanced hippocampal neuron survival *via* suppressing TIMP2-dependent MAPK pathway (Liu et al., 2018). Moreover, miR-410 overexpression decreased expression levels of TIMP2, p38, JNK and ERK proteins (Liu et al., 2018).

#### HIF

Hypoxia-inducible factor-1 (HIF-1), transcription factor, activated in response to oxygen levels fluctuations, modulates gene expression aimed at facilitating cell adaptation in hypoxic conditions (Sharp and Bernaudin, 2004; Shi, 2009). Noteworthy, hypoxic/pharmacological induction of HIF-1 *in vivo* and *in vitro* ischemic stroke models elicited neuroprotection against ischemic insult by promoting antiapoptotic mechanisms and contributing to the neuronal cell’s survival (Siddiq et al., 2005; Baranova et al., 2007). However, depending on the intensity of the injurious stimulus and duration of ischemia, HIF-1 might promote both cell survival in mild hypoxic conditions or neuron apoptosis in long-term hypoxia (Helton et al., 2005; Baranova et al., 2007). Serum samples of 52 ischemic stroke patients showed a lower miR-210 expression level, with a variable mean of miR-210 between different time points (time of admission and 3 months after stroke) and a higher HIF-1α levels, which does not change in a time-dependent manner. Increased expression levels of miR-210 and decreased HIF-1α levels exhibited a better survival rate in these patients (Rahmati et al., 2021). In OGD/R induced neuroblastoma cells microRNA-186 elicited antiapoptotic effects, by downregulating HIF-1α (Li et al., 2021b). PC12 cells exposed to OGD/R injury exhibited elevated miR-134 and HIF-1α expression levels. HIF-1α overexpression may alleviate OGD/R-induced injury, by suppressing miR-134 expression (Zhang et al., 2020a). Moreover, by inhibiting miR-134 expression, HIF-1α induces the activation of ERK1/2 and JAK1/STAT3 pathways (Zhang et al., 2020a).

#### Vascular Endothelial Growth Factor

Vascular endothelial growth factor (VEGF), a pro-angiogenic factor which modulates vasculogenesis and neoangiogenesis, presents essential properties in both physiological and pathological conditions, such as wound healing and repair, pregnancy, diabetic retinopathy, tumor growth and metastasis, and ischemic processes, myocardial infarction, and ischemic stroke (Melincovici et al., 2018; Shim and Madsen, 2018). VEGF regulates cerebral angiogenesis after stroke, promoting either restoration of blood supply after ischemic injury, or promoting BBB disruption by increasing vascular permeability (Zhang et al., 2017; Geiseler and Morland, 2018). The beneficial or deleterious effects promoted by VEGF depends on the level of expression of VEGF. For instance, an elevated VEGF expression leads to neurological deterioration, whereas an appropriate level of VEGF sustains the recovery process of brain in response to hypoxia (Zhang et al., 2021b).

Brain Microvascular Endothelial Cells (BMVEC) exposed to OGD elicited increased level of VEGF and reduced miR-150 expression. In OGD-induced BMVEC cells, downregulation of miR-150 and upregulated its predicted target, MYB induced VEGF expression, thus regulating cerebral angiogenesis after ischemic stroke (Zhang et al., 2021b). Serum samples from 78 diabetic and non-diabetic patients with ischemic stroke (acute ischemic stroke or transient ischemic attack) revealed a high level of miRNA-195-5p and miRNA-451a at 0, 24, and 72 hours after the stroke event, with low levels of BDNF and VEGF-A at the same time-points (Giordano et al., 2020).

#### Brain Derived Neurotrophic Factor

The brain derived neurotrophic factor (BDNF), crucial neurotrophic factor involved in the regulation process of synaptic transmission and brain plasticity activity, promotes neuroprotective effects in hypoxic and excitotoxic-induced neuron cell death (Degos et al., 2013; Miranda et al., 2019).

Besides transcriptional and translational regulation, BDNF expression might be regulated upon post-transcriptional level, by epigenetic mechanisms, including neuronal activity, hormones environmental factors such as exercise and stress (Metsis et al., 1993; Lubin et al., 2008; Miranda et al., 2019). The expression levels of BDNF have a high reach in hippocampus, being also detected in the cerebellum, cerebral cortex and amygdala (Hofer et al., 1990). In MCAO mice model, upregulated level of miR-191-5p was associated with disturbed angiogenesis, by inhibiting BDNF, suggesting the neuroprotective mechanisms promoted by miR-191-5p inhibition (Wu et al., 2021). In OGD-induced mouse neurons and astrocytes, inhibiting miR-128 by treatment with ARPP21 antagonistic intron exhibited up-regulation of BDNF and CREB1, therefore inhibiting apoptosis and promoting neurological recovery against ischemic stroke (Chai et al., 2021).

#### PI3K, AKT

Mounting evidence revealed the involvement of PI3K/Akt signaling pathway in cerebral ischemic/hypoxic injury, emerging new promising strategy for ischemic stroke (Zhang et al., 2018). By phosphorylating the inositol group in the plasma membrane phospholipids, PI3K/Akt pathway acts as a critical regulator of multifold cell processes, including cell growth, proliferation, coagulation, inflammation under different physiological and pathological settings (Fruman et al., 1998; Li et al., 2008).

Activation of the PI3K/Akt pathway by increasing miR-18b exhibited decreased apoptosis rate and reduced neuroinflammation in OGDR induced SH-SY 5Y cell dysfunction and MCAO mice model (Min et al., 2020). MiR-22 exhibited low expression level in cerebral I/R injury. Treatment with miR-22 mimic in MCAO rat model revealed increased levels of serum VEGF and Ang-1 and the levels of p-PI3K/PI3K and p-Akt/Akt proteins. Thus, miR-22 promoted angiogenic and neuroprotective effects in ischemia/reperfusion injury by activating PI3K/Akt signaling pathway (Wang et al., 2020b).

#### Aquaporin

Aquaporin (AQP)-4, the active regulator of water flux, poses critical role in edema formation, emerging new therapeutic targets for counteracting vascular edema in ischemic stroke (Zador et al., 2009). In this context, miR-29b, 130a and -32 were shown to repress AQP-4 (Sepramaniam et al., 2010, 2012; Wang et al., 2015b). MiR-29b overexpression promoted neuroprotection in ischemic stroke, by ameliorating BBB disruption upon ischemic stroke. Moreover, AQP-4 expression significantly decreased after miR-29b overexpression (Wang et al., 2015b, 4). Treatment of OGD-induced human astrocytoma cells injury and MCAO rat model with anti-miR-320a exhibited decreased infarct volume of cerebral ischemia, *via* upregulation of AQP1 and 4 (Sepramaniam et al., 2010).

## Conclusion

All these mechanisms are simultaneously present during I/R injury and it is hard to separate these events from each other. MiRNAs are interlinked with oxidative stress damage, inflammatory mediators production, inflammation and cell death. As a general rule, “reversing” the expression of the miRNAs involved in cerebral I/R injuries (inhibiting an over-expressed miRNA or mimicking the effect of a down-regulated miRNA) improved the outcome and studied parameters. This holds true for the majority of studies and could mean that a miRNA-centered therapeutic approach could be beneficial. Although experimental *in vivo* and *in vitro* models showed outcome improvements when analyzing one pathway and miRNA, it is very likely that in a clinical setting these strategies to be insufficient. It could be that by inhibiting one pathway, another one to over-express or that the benefit of such therapies to be clinically insignificant. Further research is needed to determine the exact roles of miRNAs and of miRNAs stimulation or inhibition in I/R injuries and to determine the most favorable candidates as treatment options.

## Author Contributions

M-AN, A-OM, C-CB, A-II, CM, MB, and C-SM were involved in the literature search and writing of the manuscript. A-OM, C-CB, and MB prepared the figure and tables. M-AN, A-OM, and A-DB performed the critical reading of the manuscript. All authors contributed to manuscript preparation and revision and reviewed the final version making the necessary changes and approved the submitted version.

## Conflict of Interest

The authors declare that the research was conducted in the absence of any commercial or financial relationships that could be construed as a potential conflict of interest.

## Publisher’s Note

All claims expressed in this article are solely those of the authors and do not necessarily represent those of their affiliated organizations, or those of the publisher, the editors and the reviewers. Any product that may be evaluated in this article, or claim that may be made by its manufacturer, is not guaranteed or endorsed by the publisher.
